# Dietary Fructose Enhances the Ability of Low Concentrations of Angiotensin II to Stimulate Proximal Tubule Na^+^ Reabsorption

**DOI:** 10.3390/nu9080885

**Published:** 2017-08-16

**Authors:** Agustin Gonzalez-Vicente, Pablo D. Cabral, Nancy J. Hong, Jessica Asirwatham, Nianxin Yang, Jessica M. Berthiaume, Fernando P. Dominici, Jeffrey L. Garvin

**Affiliations:** 1 Department of Physiology and Biophysics, School of Medicine, Case Western Reserve University, Cleveland, OH 44106, USA; pdcabral@gmail.com (P.D.C.); nxh156@case.edu (N.J.H.); jaa122@case.edu (J.A.); nxy66@case.edu (N.Y.); jessica.berthiaume@case.edu (J.M.B.); jlg5@case.edu (J.L.G.); 2 Facultad de Farmacia y Bioquímica, Universidad de Buenos Aires, Ciudad Autónoma de Buenos Aires C1113AAD, Argentina; dominici@qb.ffyb.uba.ar; 3 Facultad de Medicina, Departamento de Ciencias Fisiológicas, Universidad de Buenos Aires, Ciudad Autónoma de Buenos Aires C1121ABG, Argentina; 4 Current Address: Miromatrix Medical Inc., 10399 W 70th St, Eden Prairie, MN 55344, USA; 5 Instituto de Química y Fisicoquímica Biológicas, CONICET, Ciudad Autónoma de Buenos Aires C1113AAD, Argentina

**Keywords:** kidney, hypertension, sodium transport, salt sensitivity

## Abstract

Fructose-enriched diets cause salt-sensitive hypertension. Proximal tubules (PTs) reabsorb 70% of the water and salt filtered through the glomerulus. Angiotensin II (Ang II) regulates this process. Normally, dietary salt reduces Ang II allowing the kidney to excrete more salt, thereby preventing hypertension. We hypothesized that fructose-enriched diets enhance the ability of low concentrations of Ang II to stimulate PT transport. We measured the effects of a low concentration of Ang II (10^−12^ mol/L) on transport-related oxygen consumption (QO_2_), and Na/K-ATPase and Na/H-exchange (NHE) activities and expression in PTs from rats consuming tap water (Control) or 20% fructose (FRUC). In FRUC-treated PTs, Ang II increased QO_2_ by 14.9 ± 1.3 nmol/mg/min (*p* < 0.01) but had no effect in Controls. FRUC elevated NHE3 expression by 19 ± 3% (*p* < 0.004) but not Na/K-ATPase expression. Ang II stimulated NHE activity in FRUC PT (Δ + 0.7 ± 0.1 Arbitrary Fluorescent units (AFU)/s, *p* < 0.01) but not in Controls. Na/K-ATPase activity was not affected. The PKC inhibitor Gö6976 blocked the ability of FRUC to augment the actions of Ang II. FRUC did not alter the inhibitory effect of dopamine on NHE activity. We conclude that dietary fructose increases the ability of low concentrations of Ang II to stimulate PT Na reabsorption via effects on NHE.

## 1. Introduction

Consumption of fructose has increased dramatically in the past three decades due to addition of low-cost high-fructose corn syrup to processed foods and sugar-sweetened beverages. In European countries and the United States, calories ingested from fructose follow a normal distribution with a mean of approximately 10% of the daily caloric intake coming from this sugar [1,2,3]. This not only reduces the quality of the diet [4] but sets the second quartile at risk of ingesting at least 20% of the calories as fructose [1,2,3], primarily as sugar-sweetened beverages [3,5]. Dietary fructose causes hypertension and cardiovascular disease in humans [6,7,8,9,10,11,12,13]. It also causes hypertension in rats fed comparable amounts of fructose to those consumed by people [14,15,16]. The increases in blood pressure occur even when plasma insulin, glucose and/or lipids are within normal ranges [14,17,18].

Hypertension cannot be sustained without a renal defect or pressure natriuresis would return blood pressure to normal values. Defects in proximal tubule transport or its regulation result in changes in blood pressure [19,20]. In this segment, most of the Na enters the cells in exchange for protons by the apical Na/H exchanger type 3 (NHE3) while the remainder drives sugar, amino acid and phosphate uptake via other specific transporters. Exit of Na from the cells into the blood is mediated by Na/K-ATPase.

Ang II is a key regulator of blood pressure primarily via actions on the kidney in general [21], and the proximal tubule specifically [22]. These effects are mostly mediated via Ang II type 1 receptors (AT1R) [22] followed by activation of protein kinase C (PKC) [23,24,25,26]. AT1R antagonists ameliorate hypertension in the fructose-fed rat [27,28,29,30], indicating that Ang II signaling is involved in the pathology of this model. We previously reported that acute exposure of proximal tubules to fructose enhances the ability of a low concentration of Ang II (10^−12^ mol/L) to elevate NHE activity [16]. Such low concentrations of Ang II had no effect in the absence of fructose or in the presence of an equimolar concentration of glucose [16]. However, the effects of dietary fructose on the actions of Ang II in the proximal tubule remain to be elucidated.

The proximal tubule displays a biphasic response to Ang II. Concentrations ranging from 10^−12^ to 10^−10^ mol/L progressively stimulate transport, while concentrations greater than 10^−8^ mol/L inhibit it [31,32]. Even though renal cortical Ang II levels are not precisely known, proximal tubule Na reabsorption is stimulated by normal physiological concentrations of Ang II when animals are on a normal-salt diet [21,33]. Experiments comparing the effects of Ang II infusion on Na retention in salt-restricted vs. salt-loaded rats indicate that Ang II levels maximally stimulate Na reabsorption in rats consuming reduced-salt diets [34,35]. Taken together, these data indicate that, in rats fed normal salt, renal cortical Ang II likely range from 10^−11^ mol/L [36,37] to 10^−10^ mol/L [38,39,40,41].

When animals consume excess dietary salt systemic Ang II levels fall by about an order of magnitude [42]. Thus, plasma Ang II concentrations are around 10^−12^ mol/L when rats are fed high salt. Reductions in renal Ang II allow increased urinary Na excretion (UNaV), and thereby elimination of excess salt. If this process is blocked, salt is retained and an elevation in blood pressure is required to restore Na balance causing salt-sensitive hypertension [43,44].

Salt sensitivity of blood pressure in rodents eating fructose-enriched diets is well documented [13,16,45,46,47]. In a previous study, we have shown that rats consuming 20% fructose remain normotensive at least two weeks while on normal salt, but addition of 4% salt to the diet produces an immediate increase in blood pressure [16]. Consistently, salt restriction prevents the development of hypertension in Sprague Dawley rats given fructose-containing diets ranging from 20% in the drinking water to 66% in the chow [13,16,46]. Such data indicate that fructose interferes with salt excretion mechanisms. Given that downregulation of the renin/angiotensin system is one of the primary mechanisms by which excretion of excess salt occurs, we designed this study to evaluate whether dietary fructose increases the ability of low concentrations of Ang II to stimulate proximal tubule Na transport.

We hypothesized that 20% fructose in drinking water enhances the ability of 10^−12^ mol/L Ang II to stimulate proximal tubule Na reabsorption, and that this is due to activation of PKC and NHE rather than Na/K-ATPase activity.

## 2. Materials and Methods

Drugs and Buffers: Unless specified, all drugs and reagents were obtained from Sigma-Aldrich (St. Louis, MO, USA). The composition of all solutions used in this study is shown in Table 1. Bicarbonate-Buffered Physiological Saline was continuously bubbled at 37 °C with 95% O_2_/5% CO_2_. Osmolality was titrated with mannitol to 300 mOsmol/L. In HEPES-Buffered Physiological Saline, Acid Pulse Buffer and K-Free HEPES-Buffered Solution, the pH was titrated to 7.5 with NaOH and osmolality to 300 mOsmol/L with mannitol. The 4X Reaction Media, and the 4X Reaction Media with Ouabain contained 6 U/mL of Pyruvate Kinase (PK) and 9 U/mL Lactic Dehydrogenase (LDH). The PK/LDH enzyme mix (Sigma, St. Louis, MO, USA, P0294) was also source of 1 mmol/L KCl. Both solutions contained 0.1% of dimethyl sulfoxide (DMSO) used as an ouabain carrier. The pH-sensitive fluorescent dye 2′,7′-bis-(2-carboxyethyl)-5-(and-6)-carboxyfluorescein acetoxymethyl ester (BCECF-AM; Molecular Probes, Eugene, OR, USA) was dissolved daily in anhydrous DMSO and diluted in HEPES-Buffered Physiological Saline to reach a final concentration of 1 μmol/L.

Animals: This study was approved by the Case Western Reserve University Institutional Animal Care and Use Committee. All experiments were conducted in accordance with the National Institutes of Health Guidelines for the Care and Use of Laboratory Animals. Male Sprague Dawley rats (Charles River Breeding Laboratories, Wilmington, MA, USA) weighing ~130 g were used in all protocols. All efforts were made to minimize stress and discomfort.

***Protocol 1:*** To measure blood pressure and run a metabolic panel, 11 animals were randomly divided into 2 dietary treatments: (1) the Control which drank water purified with a Milli-Q system (Millipore Sigma, Billerica, MA, USA); and (2) FRUC which drank a 20% fructose solution. Both groups received an artificial purified diet (TestDiet, St. Louis, MO, USA, #5876) containing ~100 meq/kg of Na (0.61% if expressed as NaCl). Animals were allowed to acclimate to the facility and the diet for 4–7 days while drinking tap water. After the acclimation period animals were randomly split and assigned to either FRUC or Control groups (Day 0). From Day 6 to Day 7, animals were housed individually, to measure food and water consumption every 24 h. Final weight and blood pressure were also measured. At Day 8, animals were anesthetized with isoflurane using 100% O_2_ as a carrier and underwent terminal surgery. Blood was drawn from the thoracic aorta using a 20 Gauge needle on a 10 mL heparinized syringe. Immediately after collection pH, Na, K, Cl and Lactate were measured using a Nova Prime Blood Analyzer (Nova Biomedicals, Walthman, MA, USA). The remainder of the blood was centrifuged for collection of plasma. Plasma was aliquoted and frozen for insulin measurements. Insulin was determined using a Rat Insulin ELISA kit (MERCODIA AB, Uppsala, Sweden) according to manufacturer recommendations.

***Protocol 2:*** For all studies involving tubule dissection or suspensions, animals received regular rat chow (~100 meq/kg Na), and either: (1) tap water; or (2) 20% fructose in drinking water (FRUC). After 7–9 days of dietary treatment, the animals were anesthetized with ketamine (100 mg/kg bw IP) and xylazine (20 mg/kg bw IP), and given 2 IU heparin (IP). Only one sample, either a proximal tubules suspension or a microdissected proximal tubule was obtained per animal.

Proximal Tubule Suspensions: Proximal tubule suspensions were generated using methods similar to those we used before [48]. Briefly, rats were anesthetized and an abdominal u-shaped incision was made. The kidneys were retro-perfused from the abdominal aorta with 80 mL of Bicarbonate-Buffered Physiological Saline at 37 °C containing 1 mg/mL collagenase and 2 U/mL heparin at 0.7 mL/min.

Immediately after perfusion the kidneys were excised and rapidly cooled by immersion in Bicarbonate-Buffered Physiological Saline at 4 °C. The cortex of each kidney was gently scraped with a blade, minced and transferred to a 5 mL conical tube. Tissue was disrupted by passing it through a pipette tip and stirring on ice for 5 min. The resulting suspension was filtered through a 390 μm mesh, and the tubules recovered by centrifugation at 4 °C (100× *g* for 2 min). The tubules were rinsed, filtered through a 250 µm mesh, and recovered by centrifugation at 4 °C (80× *g* for 2 min). The final pellet was resuspended in 5–10 mL of warm, gassed Bicarbonate-Buffered Physiological Saline. After sitting for 1 min to sediment glomeruli, 3 mL of the upper suspension were taken for experiments.

Oxygen Consumption: QO_2_ was measured using methods similar to those we reported [49]. Briefly, 2 to 4 mg of protein from proximal tubule suspensions were taken to a final volume of 6 mL in the chamber of a YSI Model 5301B bath assembly (Yellow Springs Instruments, Yellow Springs, OH, USA). The chamber was equilibrated at 37 °C with a gas mix containing 95% O_2_/5% CO_2_ and then closed. The oxygen tension in the chamber was monitored using a YSI Model 5300 Biological Oxygen Monitor (Yellow Springs Instruments) attached to a PowerLab (ADInstruments, Colorado Springs, CO, USA). After stabilization of about 90 s, basal QO_2_ was recorded for 1 min, and 10^−12^ mol/L Ang II was added while QO_2_ was continuously measured. At the end of the experiment tubules were recovered by centrifugation to determine protein content. The results were expressed as nmol O_2_/mg protein/min.

Western Blotting: Western blotting of fresh samples was conducted using similar techniques as before [50]. In brief, proximal tubule suspensions were dissolved in lysis buffer (Sigma, CelLytic^TM^) containing 1% protease inhibitor cocktail (Sigma, P8340). Lysed samples rested for at least 5 min on ice and debris was removed by centrifugation (5600× *g*, 5 min, 4 °C). Protein content was determined by a colorimetric assay. Samples were loaded onto 6% polyacrylamide gels, loading equal amounts of protein on each lane. Samples from FRUC and Control were processed in pairs and loaded on the same gel, so each gel had its own control. Electrophoresis was performed at 100 V and proteins transferred to a 0.45 micron PVDF (polyvinylidene difluoride) membrane (Bio-RAD, Hercules CA, USA) overnight. After transferring, membranes were cut at about 60 kD. The upper membranes were blotted for either Na/K-ATPase α1-subunit or NHE3, while the lower parts were blotted for either β-tubulin or glyceraldehydephosphate dehydrogenase (GAPDH) as loading controls, respectively. Western blots were conducted as specified in Table 2. All incubations were at room temperature. No membrane was stripped.

Protein Detection and Densitometry: Membranes were incubated with Luminta^TM^ Classico Western HRP Substrate (Millipore, Billerica, MA, USA) and the signal detected by exposing a HyBlot CL (Denver Scientific, Holliston, MA, USA) autoradiography film to the membranes. Films were scanned in a single session using an EPSON Expression 1680 scanner with EPSON-Scan software (Settings: positive film, 16-bit greyscale, 600 dpi) and densitometry measured using Image J 1.47p software. After densitometry, the average peak-area of all experimental lanes in each membrane was calculated and the peak-areas of individual lanes were expressed as a fraction of this value. Results were expressed as Na/K-ATPase/β-tubulin ratio and as NHE3/GAPDH ratio.

Na/K-ATPase activity: The hydrolytic activity of the Na/K-ATPase was measured by coupling ADP production to 2 enzymatic reactions which produce NAD+, similar to what we have done before [51]. Briefly, an aliquot of proximal tubule suspension was rinsed with K-Free HEPES-Buffered Solution and placed on an inverted microscope. Using micro tweezers, 2 tubules totaling not less than 0.5 mm were transferred to a 0.5 mL “safe-lock”-Eppendorf tube containing 30 µL of 0.7% octyl glucoside (Sigma—#30887) and either vehicle or Ang II. Tubules were incubated on ice between 10 and 15 min, and then 13 µL of 4X Reaction Media were added to all tubes but the ouabain ones which received 4X Reaction Media with Ouabain. Samples were incubated at 37 °C for 30 min. The reaction was stopped by adding 53 µL of 0.5 mol/L HCl to each tube and then incubating the mix for 15 min at 37 °C. Finally, the whole reaction volume was transferred to a 2 mL Eppendorf tube containing 1 mL of 6 mol/L KOH and incubated at 60 °C for 20 min in the dark. All samples were measured on a Hitachi F2700 spectrofluorometer, exciting at 366 nm and collecting light at 455 nm. A calibration curve was obtained by adding 0.5 to 3 nmol of ADP to different tubes, which were processed under the same conditions as the samples. Linear regression of the data provided a slope used to calibrate the assay. Na/K-ATPase activity was expressed as pmol ATP/mm/min.

Isolation of Proximal Tubules: The abdominal cavity was opened and the left kidney bathed in ice-cold 150 mmol/L NaCl. The kidney was removed and placed in ice-cold HEPES-Buffered Physiological Saline at 4 °C. Coronal slices were cut and proximal tubules were isolated from the cortex using microforceps under a stereomicroscope at 4 to 10 °C. Tubular segments ranging from 0.7 to 1.0 mm were then transferred to a temperature-regulated chamber and perfused using concentric glass pipettes at 37 ± 1 °C as we have previously described [16,50].

Measurement of NHE Activity Using the NH_4_Cl Acid Pulse: The intracellular fluorescence detection system was set up on a Nikon Diaphot inverted microscope (Nikon, Japan). Proximal tubules were loaded with 1 μM BCECF-AM in the basolateral bath at 37 ± 1 °C for 5 min and then washed for 10 min in dye-free HEPES-Buffered Physiological Saline. BCECF was excited alternately at 490 and 450 nm and emitted fluorescence measured using a 510 nm dichroic mirror. Fluorescent images were taken utilizing a 40X oil immersion objective and a Coolsnap HQ digital camera (Photometrics, Tucson, AZ, USA), and ratiometric measurements (490/450) were recorded using Metafluor version 7 imaging software (Universal Imaging, Downington, PA, USA).

After the dye-free washing period measurements were taken once every 2 s for 1 min. The basolateral bath solution was then switched to the Acid Pulse Buffer. After 20 s, the basolateral bath was exchanged back to HEPES-Buffered Physiological Saline. This procedure causes an initial intracellular alkalinization followed by intracellular acidification. The initial rate of intracellular pH recovery that follows was taken as a measurement of NHE activity and quantified as fluorescent units per s (AFU/s). Different compounds of interest were then added to the basolateral bath as indicated in the Results section. After a 10 min re-equilibration period, the NH_4_Cl acid pulse was repeated. The recovery rates in both periods were compared.

Systolic Blood Pressure: Blood pressure was measured non-invasively in conscious animals using the CODA tail-cuff blood pressure system (Kent Scientific, Torrington, CT, USA). Animals were trained on Days 0, 2 and 4 of the dietary treatment and the final measurement was taken at 9:00 a.m. on Day 7.

Statistics: All data were analyzed using R, version 3.2.3 (R Foundation for Statistical Computing, Vienna, Austria. URL https://www.R-project.org/). Means were compared using unpaired 2-tailed Student t-tests. All *p* values *<* 0.05 were considered significant. Results are expressed as the arithmetic mean ± the standard error of the mean of each group, or by the difference between two means ± the standard error of that difference.

## 3. Results

A metabolic panel of this model is presented in Table 3. Animals on both diets gained weight at the same rate and ingested similar calories. However, the FRUC group ate ~30% less food. There were no differences in weight or systolic blood pressure after seven days of treatment. Fructose feeding did not affect the main electrolytes Na, K and Cl, nor the acid–base balance as measured by lactate and pH. Insulin levels of both groups were within normal range. Together, these data indicate that consumption of 20% fructose in the drinking water for seven days does not cause metabolic syndrome.

**Figure 1:**
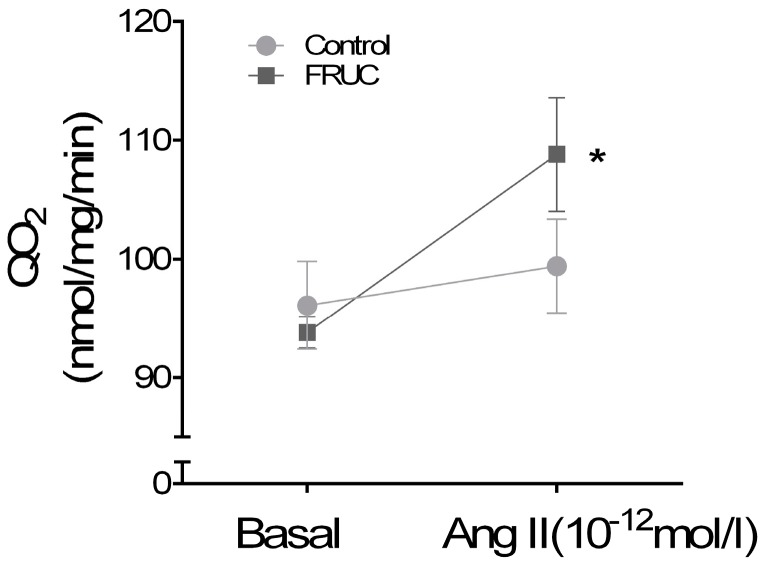
Effect of 10^−12^ mol/L Ang II on QO_2_ in proximal tubule suspensions from rats consuming either 20% fructose (FRUC) or tap water (Control). Ang II stimulated QO_2_ in FRUC (*n* = 12) but not in Controls (*n* = 12). In FRUC, * indicates *p <* 0.01 for 10^−12^ mol/L Ang II vs. basal.

We started testing our hypothesis by studying whether a low concentration of Ang II affects transport in proximal tubules suspensions. We found that Ang II (10^−12^ mol/L) stimulated QO_2_ by 14.9 ± 3 nmol/mg/min (*p* < 0.01, *n* = 12) in suspensions from FRUC. In contrast, QO_2_ was 96 ± 4 nmol/mg/min before Ang II and 99 ± 6 nmol/mg/min afterwards in Control suspensions (Figure 1). Thus, although basal transport rates were similar in both groups, Ang II (10^−12^ mol/L) only enhanced QO_2_ in suspensions from the rats on the fructose diet. To show that the lack of an effect of 10^−12^ Ang II in control suspensions was not due to a complete inability to respond to Ang II, we tested 10^−9^ Ang II and found no difference between groups in total QO_2_ (FRUC 116 ± 5 nmol/mg/min vs. 115 ± 7 nmol/mg/min in Control; *n* = 12).

**Figure 2:**
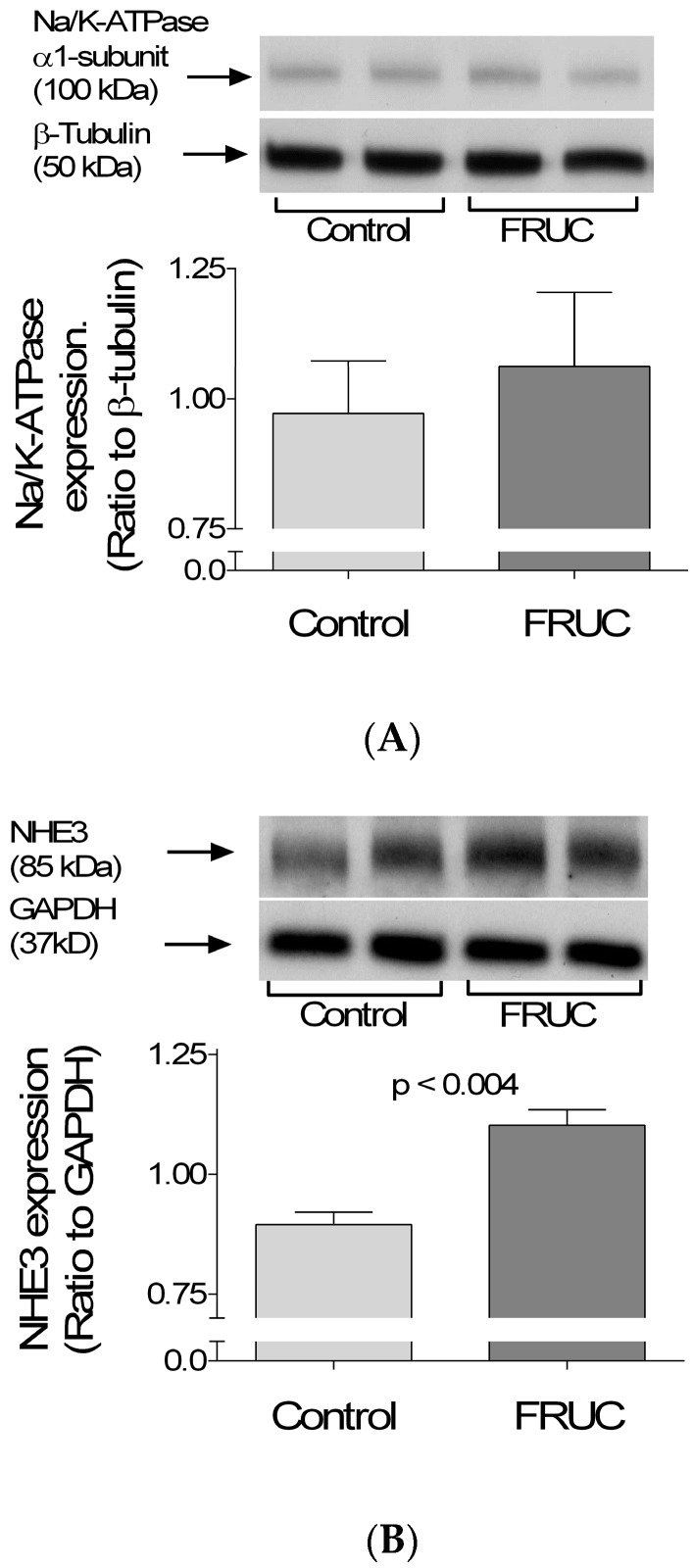
Effect of drinking 20% fructose (FRUC) or tap water (Control) on the expression of: (**A**) Na/K-ATPase; and (**B**) NHE3 in proximal tubule suspensions, as measured by Western blots. Results are relative to β-tubulin and GAPDH, respectively.

Transport rates are determined in part by transporters’ abundance. Thus, we assessed protein levels of the two main Na transporters in proximal tubules, the α1 subunit of Na/K-ATPase and NHE3 by Western blots. We found no difference in the α1-subunit/β-tubulin ratio of tubules from the two dietary groups (1.06 ± 0.1 vs. 0.97 ± 0.10; Figure 2A). In contrast, we found that the NHE3/GAPDH optical density ratio was 1.10 ± 0.03 in tubules from FRUC and 0.90 ± 0.03 in controls (Δ + 19 ± 3%; *p* < 0.004; Figure 2B).

**Figure 3:**
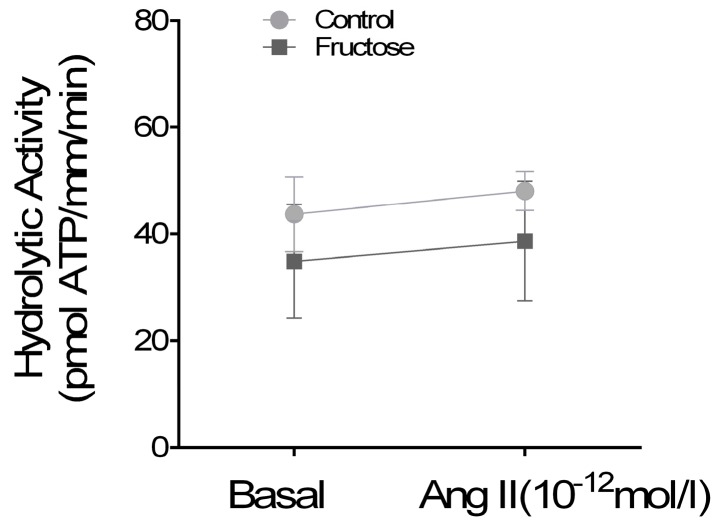
Effect of 10^−12^ mol/L Ang II on Na/K-ATPase hydrolytic activity, in permeabilized proximal tubule from rats consuming either 20% fructose (FRUC) or tap water (Control).

To assess intrinsic changes in transporter function, we measured Na/K-ATPase and NHE3 activities. Na/K-ATPase was assessed by the ATP hydrolytic activity of permeabilized tubules, where Na gradients secondary to enhanced apical entry are negligible. Under these conditions, Ang II (10^−12^ mol/L) did not significantly enhance Na/K-ATPase activity in tubules from either group (Figure 3). In FRUC tubules, Na/K-ATPase was 35 ± 11 pmol ATP/mm/min without Ang II and 39 ± 11 pmol ATP/mm/min with Ang II (*n* = 7). In controls, the values were 44 ± 7 pmol ATP/mm/min without Ang II and 48 ± 4 pmol ATP/mm/min with Ang II (*n* = 7). Ang II (10^−9^ mol/L) used as a positive control stimulated Na/K-ATPase activity to the same extent in tubules from both groups (63 ± 15 pmol ATP/mm/min in FRUC vs. 70 ± 9 pmol ATP/mm/min in controls; *n* = 7). These results show that Na/K-ATPase is not affected by dietary fructose.

**Figure 4:**
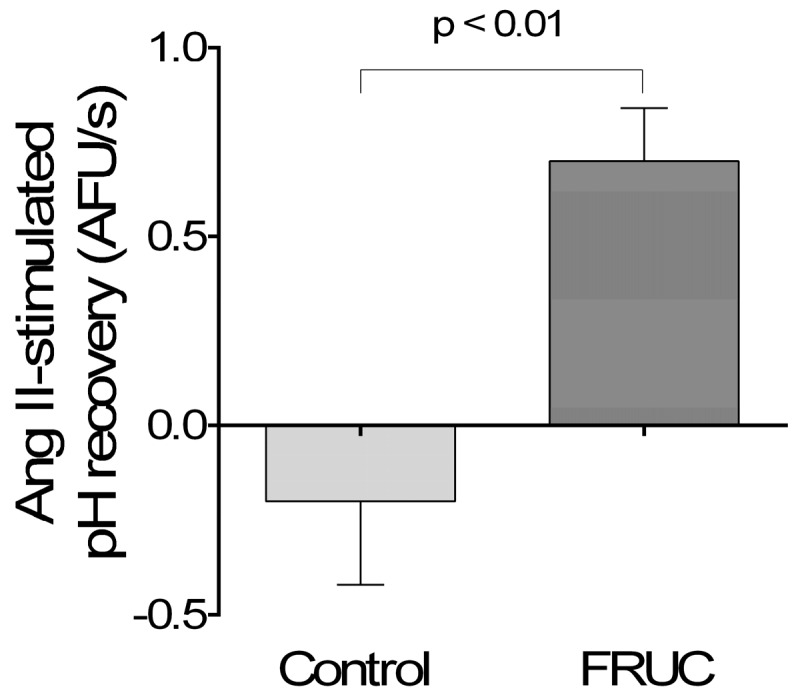
Effect of 10^−12^ mol/L Ang II on pH recovery after an NH_4_ pulse in proximal tubules isolated from rats consuming either 20% fructose (FRUC) or tap water (Control).

NHE3 activity was assessed by pH recovery in isolated perfused proximal tubules. Addition of 10^−12^ mol/L Ang II had no significant effect on pH recovery in Control tubules which recovered at a rate of 2.0 ± 0.2 arbitrary fluorescent units (AFU)/s without and at 1.8 ± 0.1 with 10^−12^ mol/L Ang II (Δ − 0.2 ± 0.2 AFU/s). In the FRUC tubules, Ang II increased recovery by 0.7 ± 0.1 AFU/s (*p* < 0.01; *n* = 5; Figure 4). These results show that a low concentration of Ang II only stimulated NHE in tubules from rats receiving fructose in the drinking water.

**Figure 5:**
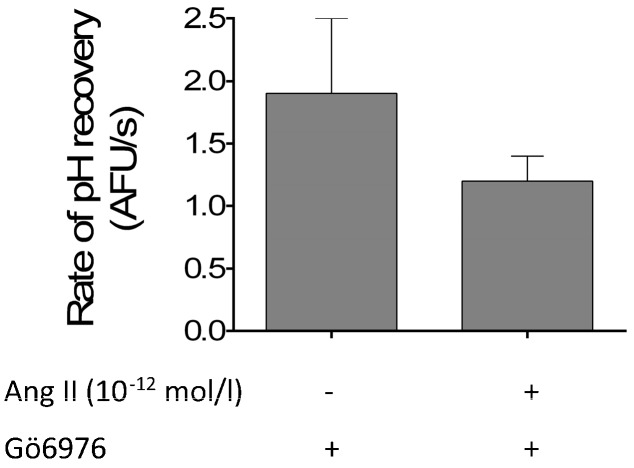
Effect of 10^−12^ mol/L Ang II on pH recovery after an NH_4_ pulse in the presence of the PKC inhibitor Gö6976 in proximal tubules from rats consuming 20% fructose.

We next studied whether changes in Ang II signaling could be affecting NHE3 activity. When PKCα/β1 were inhibited, the pH recovery rate in the presence of Ang II (10^−12^ mol/L) in tubules from animals on fructose was 1.2 ± 0.2 AFU/s, not different from the 1.9 ± 0.6 AFU/s in its absence (*n =* 5; Figure 5). These data indicate that, upon PKCα/β1 inhibition, FRUC tubules lack the ability to increase pH recovery in response to 10^−12^ mol/L Ang II. This concentration of Ang II has no effect on pH recovery in controls (Figure 4).

**Figure 6:**
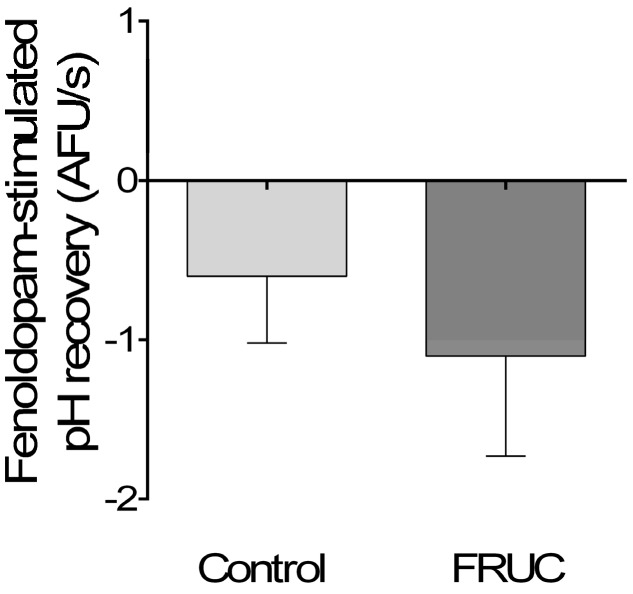
Effect of 10^−6^ mol/L of the dopamine 1 receptor agonist fenoldopam on pH recovery after an NH_4_ pulse in proximal tubules from rats consuming either 20% fructose (FRUC) or tap water (Control).

Impairment of inhibitory signals could be augmenting a positive response to Ang II. Thus, we explored whether the response to natriuretic factors was altered by fructose by measuring the effect of the dopamine receptor 1 (D1) agonist fenoldopam on NHE3 activity. We found that, in tubules from FRUC rats, fenoldopam (10^−6^ mol/L) reduced the rate of pH recovery by 1.1 ± 0.6 AFU/s (*n =* 7). This value was not significantly different from the recovery rate in controls after the D1 agonist of 0.6 ± 0.4 AFU/s (*n =* 5; Figure 6). This indicates that the enhanced sensitivity to Ang II is not mediated by an altered response to a natriuretic factor.

## 4. Discussion

We previously reported that 20% fructose in the drinking water predisposes animals to salt-sensitive hypertension, and that acute exposure to fructose increases the sensitivity of control proximal tubules to Ang II [16]. In the present study, we explored whether dietary fructose enhances the ability of low concentrations of Ang II (10^−12^ mol/L) to augment proximal tubule Na reabsorption and the transporters involved. We also characterized the model showing that a moderately enriched fructose diet does not cause overt metabolic syndrome within seven days. Thus, it is important to distinguish the model presented here from models with longer-term treatments (≥2 weeks) used to induce metabolic syndrome [52].

To test our hypothesis, we first measured whether a low concentration of Ang II (10^−12^ mol/L) stimulated QO_2_ by proximal tubule suspensions from rats given either 20% fructose or tap water. We found that a low Ang II (10^−12^ mol/L) increased QO_2_ by proximal tubules from rats given 20% fructose in their drinking water for a week but not in control suspensions.

QO_2_ can be used as a rapid screening tool for changes in transcellular Na transport because proximal tubules produce ATP via aerobic metabolism, and Na/K-ATPase uses 60–70% of the ATP supply to drive Na across the basolateral membrane. Thus, our data indicate that a 20% fructose diet causes low concentrations of Ang II to stimulate Na reabsorption.

One mechanism by which the response of Na reabsorption to a low concentration of Ang II could be enhanced is by increased expression of Na transporters. Consequently, we measured the expression of Na/K-ATPase and NHE3, which mediate Na exit from the cell across the basolateral membrane and most Na entry across the apical membrane, respectively. Our data indicate that a 20% fructose diet alters NHE3 but not Na/K-ATPase expression after one week. However, it is important to note that total transporter expression does not necessarily correlate with activity. Even more accurate measurements of transporter expression, such as the number of units in the plasma membrane, should be taken with caution when estimating transport rates. Thus, the modest increase in NHE3 reported here may or may not alter actual Na reabsorption in vivo.

To circumvent these problems and directly address transport, we measured Na/K-ATPase and NHE activities. We found that dietary fructose did not alter basal ouabain-sensitive ATP hydrolysis, a measure of Na/K-ATPase activity. Furthermore, a low concentration of Ang II did not affect Na/K-ATPase activity in either FRUC or control suspensions. These data indicate that dietary fructose does not directly alter Na/K-ATPase activity or the ability of Ang II to regulate it. These experiments also show a trend of lower Na/K-ATPase activities in the FRUC group. Even though this difference was not significant, a shift in Na/K-ATPase activity may represent the initiation of a pressure natriuretic response [53]. This counter regulatory mechanism could be buffering the enhanced sensitivity to Ang II to prevent elevations in blood pressure. Finally, a limitation of our results is that we did not study whether dietary fructose alters the K_1/2_ for either Na or K. Given that intracellular Na is near the K_1/2_ of the Na/K-ATPase for Na, a change in this parameter could alter activity. However, while Ang II has been reported to increase maximum Na/K-ATPase activity [54], there are no reports to our knowledge that it alters the K_1/2_ for Na. It is unlikely that an increase in the affinity for K could affect Na/K-ATPase activity under normal physiological circumstances because K is nearly always saturating.

While assessing NHE3 activity, we found that a low concentration of Ang II increased pH recovery in proximal tubules from FRUC rats but not in controls. These results are the first to show such an effect, and likely explain the QO_2_ data. Examining these data, one might well ask how tubules from FRUC increase QO_2_ and NHE3 activity in response to Ang II (10^−12^ mol/L) without elevating Na/K-ATPase activity. The answer to this apparent discrepancy is rather simple. The Na/K-ATPase experiments were designed to test direct actions of Ang II on this transporter. Thus, ATP hydrolytic activity was measured in permeabilized tubules in which intracellular Na (Na_i_) was equilibrated with the extracellular media. The corollary of the data is that the increase in Na/K-ATPase activity necessary to support higher transport rates in FRUC is not achieved by a direct effect of Ang II on this transporter; instead, an elevated intracellular Na secondary to an increase in NHE activity drives the Na/K-ATPase. This explanation is supported by the literature in which stimulation of proximal tubules with Ang II triggers transient increases in Na_i_ of up to 8 mmol/L [32,55]. Such an increase in Na_i_ would be expected to double the resting levels of about 10 mmol/L [32], and elevate Na/K-ATPase activity near to its maximum velocity [49].

Our data showing that dietary fructose enhances the sensitivity of proximal tubules to Ang II is novel, and the present study is the first addressing the chronic effect of fructose consumption on proximal tubule transport. However, the acute effects of fructose on tubular transport have been measured previously. We reported that short-term incubation with millimolar concentrations of fructose increase NHE activity in response to 10^−12^ mol/L Ang II in isolated perfused proximal tubules [16]. Another group reported that millimolar concentrations of fructose increased bicarbonate transport in proximal tubules using micropuncture [56]. In both reports, the effects of fructose were unique and not shared by other hexoses [16,56].

Urinary and plasma fructose concentrations correlate with fructose intake [57,58,59] leading to elevated concentrations in the environment surrounding proximal tubules. The proximal tubule reabsorbs ~70% of filtered water which would be expected to increase the intraluminal concentration of fructose by a factor of 3–4 compared to plasma. After ingesting a 20% fructose solution, the urinary concentration of fructose can reach up to 40 mmol/L in humans [60]. Based on this, the fructose concentration in the proximal tubule can be back calculated to 3–4 mmol/L, comparable to concentrations that increase transport in vitro [16,56]. This suggests that the effects of a 20% fructose diet are likely due to fructose *per se* in the proximal tubule.

In our current work, we observed differences in transport rates in tubular suspensions and isolated perfused tubules, preparations in which variables such as arterial pressure, renal hemodynamics, plasma signaling molecules and even fructose itself have been eliminated. This necessarily means that the effects of fructose are not limited to short postprandial periods when fructose concentrations in plasma and urine are elevated. Thus, the effects of fructose are long-lasting and do not depend on acute changes in circulating factors or intact renal nerves and architecture.

The mechanisms by which dietary fructose enables low concentrations of Ang II to stimulate proximal tubule Na reabsorption are not completely understood. We found that the PKC α/β_1_ inhibitor Gö6976 blunted the ability of a low concentration of Ang II to stimulate NHE activity in proximal tubules from rats fed 20% fructose. Thus, PKC α or β_1_ likely are crucial to the actions of dietary fructose. Classical PKC isoforms (α, β, γ), which are diacylglycerol- and Ca-dependent, have been shown to mediate the stimulation of fluid and bicarbonate reabsorption caused by Ang II [23,24,25,26]. Activation of this family of kinases has been reported to increase in several tissues from fructose-fed rats [61,62]. Proximal tubules generate diacylglycerols from fructose via dihydroacetone-phosphate [63,64,65,66]. Thus, dietary fructose may be increasing diacylglycerol formation, which in turn primes PKC such that lower concentrations of Ang II can activate it and stimulate transport.

In contrast to the effect on NHE activity, dietary fructose does not allow a low concentration of Ang II to stimulate Na/K-ATPase activity. These results appear to be consistent with the literature, as PKC has been reported to have disparate effects on this transporter [67]. Early metabolic studies in proximal tubule suspensions showed that phorbol esters first stimulate and then inhibit ouabain-sensitive QO_2_. The stimulatory phase was inhibited by amiloride, and when Na_i_ was equalized with amphotericin B the inhibition phase was still observed. The authors concluded that the initial stimulation of Na/K-ATPase was secondary to an increase in Na_i_ and the inhibitory effect was dependent on PKC [68]. Other studies demonstrated that the regulation of Na/K-ATPase by PKC is also modulated by PKA and Na_i_ [67,69,70]; and phorbol esters, which stimulate classical PKCs, inhibit Na/K-ATPase activity [70].

Finally, we tested whether alterations in natriuretic signaling contribute to the enhanced sensitivity to Ang II. We measured whether a 20% fructose diet affects dopamine signaling mediated by D1s because dopamine is a major regulator of proximal tubule transport [71,72] and D1s directly interact with the AT1R counteracting their actions [73,74]. Thus, a reduction in D1 signaling would result in less inhibition of the stimulatory effect of Ang II. Our results indicate D1 signaling is not impaired in this model; this is of particular importance since the interaction between D1 and AT1R occurs at the receptor level [75], which reinforces the idea that the changes in sensitivity to Ang II are due to downstream signaling.

In summary, consumption of 20% fructose in drinking water for one week causes an increase in transport rates by the proximal tubule in response to low concentrations of Ang II that is associated with intracellular pathways involving lipid-dependent PKC isoforms.

## 5. Conclusions

In industrialized countries, the average fructose consumption represents nearly 10% of the total caloric intake [1,2,3]. This sets the second quartile at risk of ingesting 20% or more of total calories as fructose [1,2,3]. The increase in the incidence of hypertension over the last 40 years mirrors the increase in dietary fructose and salt. Our present study suggests that part of the increase in blood pressure associated with fructose consumption is due to an enhanced Ang II sensitivity in proximal tubules. This may provide an explanation for why Ang II receptor blockers frequently reduce blood pressure to a greater extent than Ang II converting enzyme inhibitors. It also suggests that reducing dietary fructose in hypertensive patients may be comparable to reducing salt intake, with the added benefits of being easier for patients to reduce fructose intake over salt, given that better substitutes are available.

## Figures and Tables

**Table 1:** Solutions and Buffers.

		Bicarbonate- Buffered Physiological Saline	HEPES- Buffered Physiological Saline	K-Free HEPES-Buffered Solution	Acid Pulse Buffer	4X Reaction Media	4X Reaction Media with Ouabain
NaHCO_3_	(mmol/L)	25.0	-	-	-	-	-
HEPES	"	-	10.0	10.0	10.0	-	-
Imidazole	"	-	-	-	-	200.0	200.0
NaCl	"	114.0	130.0	130.0	120.0	320.0	320.0
KCl	"	4.0	4.0	-	4.0	120.0	-
Na_2_HPO_4_	"	2.1	2.5	2.5	2.5	-	-
NaH_2_PO_4_	"	0.4	-	-	-	-	-
Mg SO_4_	"	1.2	1.2	1.2	1.2	20.0	20.0
Ca(Lactate)_2_	"	2.0	2.0	2.0	2.0	-	-
Na_3_Citrate	"	1.0	1.0	1.0	1.0	-	-
DL-alanine	"	6.0	6.0	6.0	6.0	-	-
Glucose	"	5.5	5.5	5.5	5.5	-	-
NH_4_Cl	"	-	-	-	10.0	-	-
EGTA	"	-	-	-	-	2.0	2.0
Na_2_ATP	"	-	-	-	-	20.0	20.0
NADH	"	-	-	-	-	4.0	4.0
Ascorbic Acid	"	-	-	-	-	4.0	4.0
PEP	"	-	-	-	-	40.0	40.0

**Table 2:** Antibodies and Blotting Conditions.

Antibody	Provider	Catalog	Source	Blocking	Conditions		
		Number		Buffer	Dilution	Buffer	Time
**NHE3**	Abcam	ab95299	Rabbit	5% BSA	1:1000	5% BSA	2 h
**α1-Na/K-ATPase**	Cell Signaling	#3010	Rabbit	5% Milk	1:5000	5% Milk	2 h
**β-tubulin**	Abcam	ab6046	Rabbit	5% Milk	1:10,000	5% Milk	2 h
**GAPDH-HRP**	Abcam	ab9485	-	5% BSA	1:15,000	5% BSA	2 h
**2ry anti-Rabbit-HRP**	GE Healthcare	NA9340V	Donkey	-	1:2500	5% BSA	1 h

* BSA: Bovine Serum Albumin, Milk: non-fat dehydrated bovine milk.

**Table 3:** Metabolic Panel.

		**Control (*n = 5*)**		**Fructose (*n = 6*)**		**Change**	**T test**
		**Mean**	**SEM**	**Mean**	**SEM**		
**Caloric Intake**	**(kcal/24 h)**	69.9	6.0	65.5	3.2	=	*p < 0.51*
**Weight Gain**	**(g/24 h)**	9.4	1.3	9.2	2.4	=	*p < 0.94*
**Fluid Intake**	**(mL/24 h)**	29.2	4.6	25.8	3.1	=	*p < 0.55*
**Food Intake**	**(g/24 h)**	17.4	1.5	12.0	0.5	↓	*p < 0.01*
**Final Weight**	**(g)**	236	7	232	10	=	*p < 0.72*
**Systolic BP**	**(mmHg)**	130	11	147	6	=	*p < 0.18*
		**Control**		**Fructose**		**Change**	**T test**
		**Mean**	**SEM**	**Mean**	**SEM**		
**pH**		7.42	0.01	7.38	0.03	=	*p < 0.18*
**Na**	**(mmol/L)**	136.6	0.6	137.5	0.5	=	*p < 0.31*
**K**	**(mmol/L)**	3.8	0.2	3.7	0.1	=	*p < 0.73*
**Cl**	**(mmol/L)**	106.4	0.5	106.0	0.3	=	*p < 0.55*
**Lactate**	**(mmol/L)**	1.02	0.14	1.03	0.07	=	*p < 0.98*
**Insulin**	**(µg/mL)**	0.38	0.14	0.36	0.15	=	*p < 0.93*
